# The emergence of multi-drug resistant and virulence gene carrying *Escherichia coli* strains in the dairy environment: a rising threat to the environment, animal, and public health

**DOI:** 10.3389/fmicb.2023.1197579

**Published:** 2023-07-13

**Authors:** Muhammad Shoaib, Zhoulin He, Xiang Geng, Minjia Tang, Ruochen Hao, Shengyi Wang, Ruofeng Shang, Xuehong Wang, Hongjuan Zhang, Wanxia Pu

**Affiliations:** Key Laboratory of New Animal Drug Project, Gansu Province/Key Laboratory of Veterinary Pharmaceutical Development, Ministry of Agriculture and Rural Affairs, Lanzhou Institute of Husbandry and Pharmaceutical Sciences of CAAS, Lanzhou, China

**Keywords:** antibiotic resistance genes (ARGs), antimicrobial resistance, multi-drug resistance, virulence associated genes, *Escherichia coli*, dairy environment

## Abstract

*Escherichia coli* is a common inhabitant of the intestinal microbiota and is responsible for udder infection in dairy cattle and gastro-urinary tract infections in humans. We isolated *E. coli* strains from a dairy farm environment in Xinjiang, China, and investigated their epidemiological characteristics, phenotypic and genotypic resistance to antimicrobials, virulence-associated genes, and phylogenetic relationship. A total of 209 samples were collected from different sources (feces, slurry, water, milk, soil) and cultured on differential and selective agar media (MAC and EMB). The presumptive identification was done by the VITEK2 system and confirmed by 16S rRNA gene amplification by PCR. Antimicrobial susceptibility testing was done by micro-dilution assay, and genomic characterization was done by simple and multiplex polymerase chain reaction (PCR). A total of 338 *E. coli* strains were identified from 141/209 (67.5%) of the samples. Most of the *E. coli* strains were resistant to sulfamethoxazole/trimethoprim (62.43%), followed by cefotaxime (44.08%), ampicillin (33.73%), ciprofloxacin (31.36%), tetracycline (28.99%), and a lesser extent to florfenicol (7.99%), gentamicin (4.44%), amikacin (1.77%), and fosfomycin (1.18%). All of the strains were susceptible to meropenem, tigecycline, and colistin sulfate. Among the resistant strains, 44.4% were identified as multi-drug resistant (MDR) showing resistance to at least one antibiotic from ≥3 classes of antibiotics. Eighteen out of 20 antibiotic-resistance genes (ARGs) were detected with *sul2* (67.3%), *bla_TEM_* (56.3%), *gyrA* (73.6%), *tet*(B) (70.4%), *aph(3)-I* (85.7%), *floR* (44.4%), and *fosA3* (100%, 1/1) being the predominant genes among different classes of antibiotics. Among the virulence-associated genes (VAGs), *ompA* was the most prevalent (86.69%) followed by *ibeB* (85.0%), *traT* (84.91%), *ompT* (73.96%), *fyuA* (23.1%), *iroN* (23.1%), and *irp2* gene (21.9%). Most of the *E. coli* strains were classified under phylogenetic group B1 (75.45%), followed by A (18.34%), C (2.96%), D (1.18%), E (1.18%), and F (0.30%). The present study identified MDR *E. coli* strains carrying widely distributed ARGs and VAGs from the dairy environment. The findings suggested that the dairy farm environment may serve as a source of mastitis-causing pathogens in animals and horizontal transfer of antibiotic resistance and virulence genes carrying bacterial strains to humans via contaminated milk and meat, surface water and agricultural crops.

## Graphical Abstract

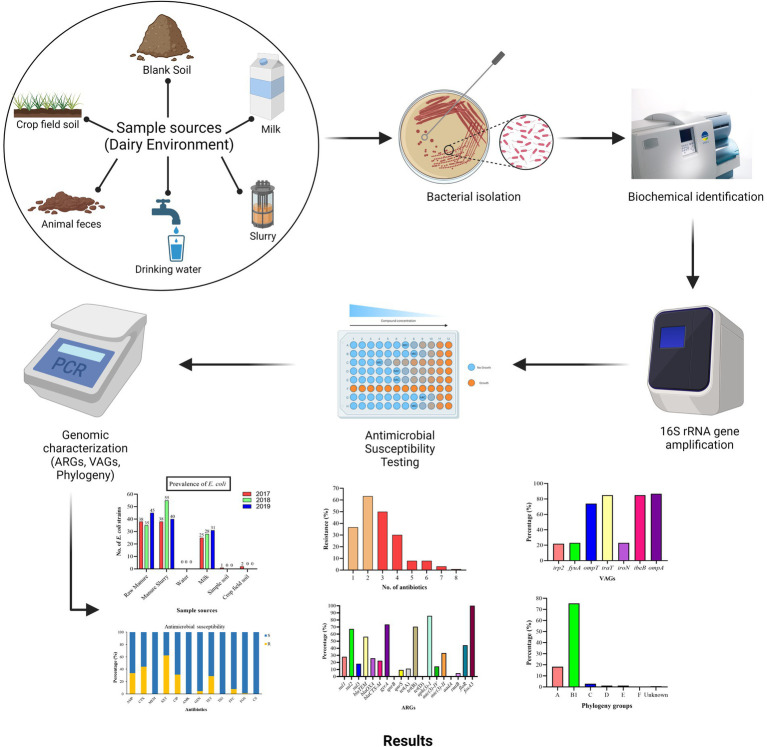

## 1. Introduction

*Escherichia coli* (*E. coli*) is an opportunistic and common inhabitant of the intestinal microbiota of animals as well as humans (He et al., 2019). Moreover, *E. coli* is also the most common organism responsible for causing udder infection in animals (Cheng et al., 2019) and bloodstream infections in humans (Jara et al., 2021). The use of antibiotics to prevent disease and promote the health of growing animals remains an integral part of livestock farming. It has been 50 years since antibiotic-supplemented feeds were first approved for livestock to improve overall health and increase the productivity of animals (Afema et al., 2018). However, the emergence and spread of pathogens resistance to multiple antibiotics has become a growing problem for veterinary medicine and public health (Murray et al., 2022). It was estimated that antimicrobial resistance results in $55 billion annual economic loss in USA (Dadgostar, 2019). The China also rank high in consumption of antibiotics for food producing animals especially in dairy sector.

Transmission of antimicrobial resistance (AMR) may occur by multiple ways, but contact with human and animal feces is the most common pathway (Graham et al., 2019). Dairy cattle act as a potential source of spread of antibiotic-resistant and zoonotic bacterial strains, especially Shiga-toxin producing *E. coli* (STEC) through the contamination of the farm environment and food products such as milk and meat, and direct contact with animals (Amézquita-López et al., 2018; Sobur et al., 2019). Animal farming, especially intensive livestock farming, plays a major role in AMR transmission between humans, animals, and the environment (Manyi-Loh et al., 2018). Due to the widespread use of antimicrobials in livestock production, livestock manure is considered a hotspot for the spread and transmission of AMR genes. Genetically diverse *E. coli* strains exist in animal manures, and their ability to survive in various ecological niches (Beattie et al., 2020). *E. coli* strains carrying *bla_CTX-M_* and *bla_CMY_* genes confer resistance to β-lactam antibiotics are frequently found in animal manure (Cookson et al., 2022). Therefore, animal manure is thought to be harming to animals *via* udder infections by environmental pathogens such as *E. coli*, humans *via* contaminated food products, and environment by using manure as fertilizer in soil or waste water (Sarowska et al., 2019). This increases the potential of antibiotic resistance genes (ARGs) to integrate into human intestinal microbiota by horizontal gene transfer mechanism (Lima et al., 2020). A better understanding of the transmission and spread of AMR, especially in areas with intensive livestock production, is important to understand. Therefore, the present study investigated the prevalence of *E. coli* in the dairy farm environment and their drug resistance characteristics. We also investigated the diversity of virulence associated genes (VAGs) responsible for pathogenicity and their distribution within phylogenetic groups.

## 2. Materials and methods

### 2.1. Sample sources and collection strategy

**Figure 1:**
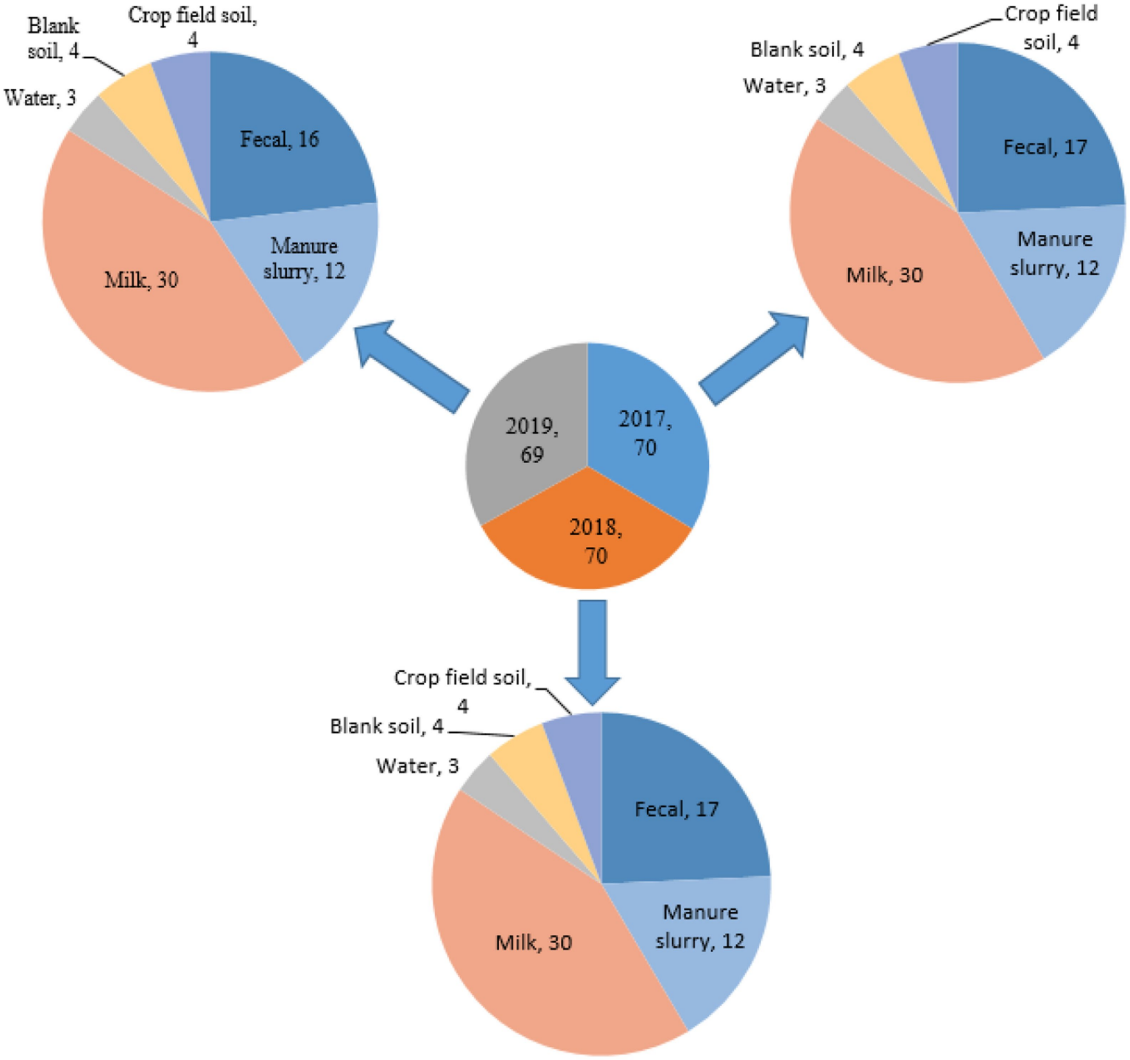
Distribution of samples collected from different sources.

A total of 209 environment samples were collected from 2017–2019 from a large dairy farm (herd size = 25,000 animals) in Xinjiang province, China. The environmental samples included were fecal samples (*n* = 50), manure slurry from a storage tank (*n* = 36), raw milk (*n* = 90), water samples from the residential area (*n* = 9), soil samples (*n* = 12), and crop field soil (*n* = 12) based on random sampling technique (Figure 1). A 50 g of manure sample was collected from the animal living area and storage tank from five different sites using a five-point mixed sampling method (Sharp et al., 2012) and stored in sterile zipper bags. Raw milk samples (10 mL) were collected and transferred to sterile falcon tubes according to the guidelines of the National Mastitis Council (Hogan and Smith, 1992). The water samples (50 mL) were collected in sterile water bottles from the residential area by randomly selecting three different water outlets. The blank and crop field soil samples were collected from different sites on farm and fodder growing fields, respectively. All the collected samples were kept at 4°C and transferred to the laboratory within 24 h for further processing.

### 2.2. Isolation and identification of *Escherichia coli*

The 25 g of fecal, manure, and soil samples were first mixed in 225 mL of phosphate-buffered saline (PBS) to solubilize them. After mixing, 1 mL of the liquid was transferred to a 10 mL LB broth tube for bacterial enrichment by incubation at 37°C with continuous mixing at 160 rpm. From each tube, 100 μL of the enrichment culture was sub-cultured on MacConkey (MAC) agar under prior mentioned incubation conditions. However, the water and milk samples were swabbed directly onto MacConkey agar and incubated at 37°C overnight. Based on colony shape and color, large, smooth, and pink colonies were picked and further streaked onto Eosin Methylene Blue (EMB) agar and incubated overnight at 37°C. The appearance of a metallic green sheen with dark center colonies on EMB agar was indicative of *E. coli* growth (Peng et al., 2022). Further, presumptive identification was done by the VITEK2 system (BioMerieux, France) (Alfinete et al., 2022) and confirmation by 16S rRNA gene amplification by PCR using primers reported previously (Liu et al., 2021). The PCR-amplified product was visualized on 1% agarose gel under the GelDoc XR system (Supplementary Figure S1). The confirmed isolates were preserved in 20% glycerol at −80°C for further analysis.

### 2.3. Detection of virulence-associated genes

The 16S rRNA-confirmed *E. coli* isolates were subjected to the identification of seven VAGs by the previously described method (Hu et al., 2022). The genomic DNA was extracted using a DNA extraction kit (Tiangen Biotech Beijing, Co., Ltd.) following the manufacturer’s guidelines. The virulence genes were identified by PCR amplification of target gene primers mentioned in Supplementary Table S1. The PCR reaction mixture (25 μL) consisted of 12.5 μL PreTaq Mix (Vazyme Biotech, China), 1 μL forward primer, 1 μL reverse primer, 1 μL genomic DNA, and 9.5 μL of deionized water under the following conditions; prior denaturation at 94°C for 5 min followed by 35 cycles of denaturation at 94°C for the 30s, annealing at varying temperatures mentioned in Supplementary Table S1 for 30s, initial extension at 72°C for 30s followed by a final extension at 72°C for 5 min. After amplification, the PCR product was run on 1% agarose gel electrophoresis at 180 V/200 mA followed by ethidium bromide staining for visualization, and images were taken under the GelDoc XR system (Supplementary Figure S2).

### 2.4. Antimicrobial susceptibility testing

The AST was done by broth micro-dilution assay following the EUCAST guidelines.^1^ Briefly, the preserved isolates were thawed at room temperature and re-suspended in LH broth by vigorous mixing (120 rpm) at 37°C for 12 h. The loopful enriched broth was streaked on MacConkey agar following the overnight incubation. The bacterial inoculum was prepared by adjusting the cell density at 5 × 10^5^ CFU/mL. The 96-well round bottom plate was used for broth dilution assay and 100 μL of Mueller Hinton (MH) broth was added from the 1st well to the 12th well with a micropipette. Next, 50 μL of prepared bacterial inoculum was added from the 1st to 11th well by keeping the 12th well as a negative control. The antibiotics were selected based on medical and veterinary use which includes trimethoprim-sulfamethoxazole (SXT), ampicillin (AMP), cefotaxime (CTX), tetracycline (TET), ciprofloxacin (CIP), gentamicin (GEN), amikacin (AMK), colistin sulfate (CS), florfenicol (FFC), meropenem (MEM), and tigecycline (TIG) were added from 1st to 10th well by keeping 11th well as a positive control. The reference strain *E. coli* ATCC 25922 was used as a quality control. The MIC (minimum concentration that inhibits visible growth of bacteria) of fosfomycin was calculated by the agar dilution method, recommended by EUCAST. The MIC of all antibiotics was evaluated by visualizing the growth in the bottom of the plate well as tinny buttons/turbidity. The MIC values were compared with standard EUCAST MIC breakpoints (Supplementary Table S2). The strains showing resistance to at least one antibiotic from ≥3 classes were classified as MDR.

### 2.5. Detection of antibiotic resistance genes

Phenotypically resistant *E. coli* strains were subjected to the detection of 20 ARGs from eight antibiotic classes (Supplementary Table S3) according to the method described previously (Yu et al., 2020). The bacterial DNA was extracted using a DNA extraction kit (Tiangen Biotech Beijing, Co., Ltd.) and used as a template for PCR amplification of 20 ARGs (listed in Supplementary Table S3). The PCR reaction mixture (25 μL) consisted of 12.5 μL PreTaq Mix (Vazyme Biotech, China), 1 μL of forward and reverse primer each, 1 μL of bacterial DNA, and 9.5 μL of deionized water. The reactions were performed under the following conditions: denaturation at 94°C for 5 min followed by 35 cycles of denaturation at 94°C for 30s, annealing at varying temperatures (see Supplementary Table S3) for 30s, and extension at 72°C for 30s, followed by a final extension at 72°C for 5 min. After amplification, the PCR product was separated on 1% agarose gel at 180 V/200 mA and stained with ethidium bromide for visualization using the GelDoc XR system.

### 2.6. Phylogenetic analysis

The phylogenetic grouping of *E. coli* strains was carried out by 2 sets of PCR using primers listed in Supplementary Table S4. The quadruple PCR reaction mixture (25 μL) consists of Premix Taq TM 12.5 μL, 1 μL of each forward and reverse primers (*chuA*, *yjaA*, *tspE4C2*), 2 μL of each *arpA* forward and reverse primers, 1.5 μL DNA template, and 1 μL dd H_2_O. PCR was carried out under the following conditions; pre-denaturation at 94°C for 4 min, 30 cycles of denaturation at 94°C for 5 s followed by annealing at 59°C for 20 s, and extension at 72°C for 5 min. The PCR reaction for group E and C identification consisted of Premix Taq TM 12.5 μL, 0.6 μL of trpBA forward and reverse primers each, 1 μL of each group-specific primer (Supplementary Table S4), 1.5 μL DNA template, and 2.8 μL dd H_2_O. In the PCR reaction solution, trpBA primers were added as an internal control. The PCR amplifications conditions were pre-denaturation at 94°C for 4 min, 30 cycles of denaturation at 94°C for 5 s, annealing at 57°C (group E) or 59°C (group C) for 20 s, and final extension at 72°C for 5 min. After the PCR amplification, the PCR product was run on 1% agarose gel and visualized under the GelDoc XR system (Supplementary Figure S3), and the phylogenetic group was identified by comparing the results with Supplementary Table S4.

### 2.7. Data analysis

The prevalence was calculated using the formula described by Thrusfield (2018).


$$Prevalence\;\left(\%\right)=\frac{No.\;ofpositiveisolates}{Totalisolates}\times 100$$

The antimicrobial susceptibility data were analyzed by descriptive statistics using Microsoft Excel. Moreover, the data for various factors such as sampling source and sampling year affecting the prevalence, AMR and virulence rates were analyzed using the Pearson’s Chi-Squared test keeping the level of significance, *α* = 5% (Zhao et al., 2021; Ma et al., 2022). *p-value* < 0.05 was considered statistically significant and vice versa. The graphical representation of data was done by GraphPad Prism version 8.2.1 and Microsoft Excel.

## 3. Results

### 3.1. Isolation of *Escherichia coli* strain from different sources

**Figure 2:**
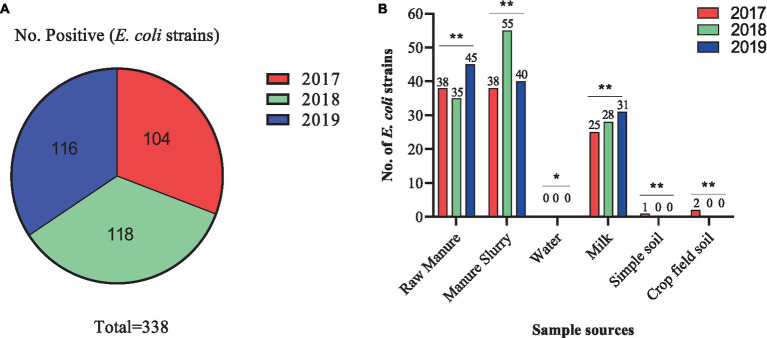
Isolation of *Escherichia coli* strains in different sampling years and sources. **(A)** Total number of *E. coli* strains isolated in different years. **(B)** Distribution of *E. coli* strains isolated from different sources in different years; **, indicate non-significant difference (*p* > 0.05); *, indicate not applicable; ***, indicate significant difference (*p* < 0.05).

A total of 209 samples were collected from different sites of dairy environment including fecal samples (*n* = 50), manure slurry from the storage tank (*n* = 36), raw milk (*n* = 90), water samples (*n* = 9), soil samples (*n* = 12), and crop field soil (*n* = 12) samples. In total, 534 suspected *E. coli* strains were isolated from 141/209 (67.5%) samples based on colony characteristics. Subsequently, 338 *E. coli* strains were confirmed by 16S rRNA gene amplification. The isolation rates were comparable over the years, with 30.8% (104/338) in 2017, 34.9% (118/338) in 2018, and 34.3% (116/338) in 2019 (Figure 2A). Overall, most of the *E. coli* strains were isolated from manure slurry (39.3%, 133/338), followed by fecal samples (34.9%, 118/338), raw milk (24.8%, 84/338), crop field soil (0.59%, 2/338), and least from blank soil (0.29%, 1/338). However, none of the *E. coli* strains was isolated from water samples (Figure 2B). In 2019, a higher number of *E. coli* strains were isolated from fecal and milk samples compared to other sampling years while more *E. coli* strains were isolated from slurry samples in 2018. Moreover, only 1 and 2 strains were isolated in 2017 from blank and crop field soil samples, respectively, while none in 2018 and 2019 (Figure 2B).

### 3.2. Antimicrobial susceptibility of *Escherichia coli* strains

**Figure 3:**
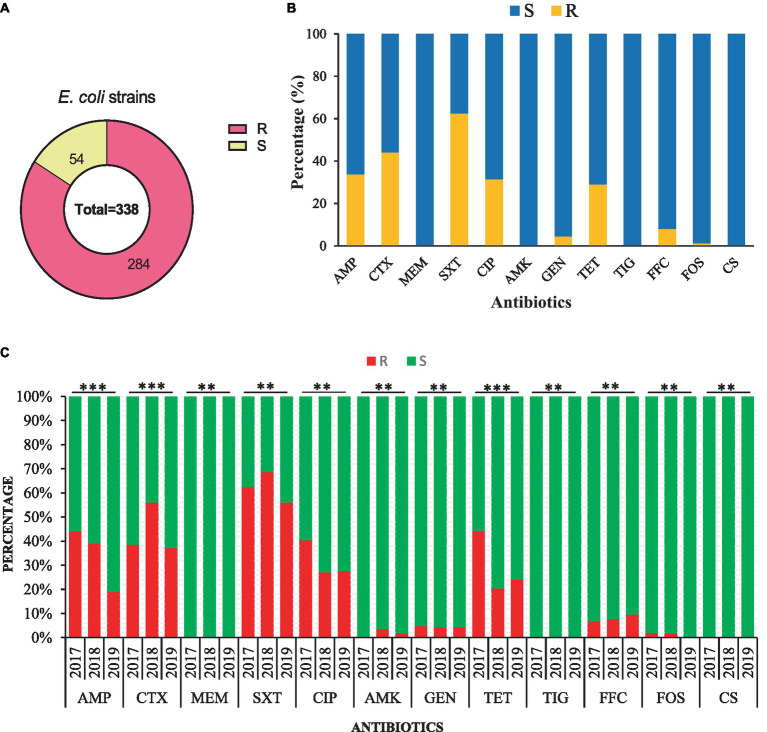
Antimicrobial susceptibility of the 338 *E. coli* strains isolated from the dairy environment. **(A)** The overall resistant (R) and susceptible (S) *E. coli* strains. **(B)** The overall antimicrobial susceptibility of 338 *E. coli* strains against individual antibiotic tested. **(C)** The comparison of antimicrobial susceptibility of *E. coli* strains from different sampling years. AMP, ampicillin; CTX, cefotaxime; MEM, meropenem; SXT, trimethoprim-sulfamethoxazole; CIP, ciprofloxacin; AMK, amikacin; GEN, gentamicin; TET, tetracycline; TIG, tigecycline; FFC, florfenicol; FOS, fosfomycin; CS, colistin sulfate; **, indicate non-significant difference (*p* > 0.05); *, indicate not applicable; ***, indicate significant difference (*p* < 0.05).

The AST of 338 *E. coli* strains showed that 284/338 (84.0%) were resistant to at least one antibiotic and 54/338 (26.0%) were susceptible strains (Figure 3A). Most of the strains were resistant to trimethoprim/ sulfamethoxazole (62.43%, 211/338), followed by cefotaxime (44.08%, 149/338), ampicillin (33.73%, 114/338), ciprofloxacin (31.36%, 106/338), tetracycline (28.99%, 98/338), and less to florfenicol (7.99, 27/338), gentamicin (4.44%, 15/338), amikacin (1.77%, 6/338), and fosfomycin (1.18%, 4/338). All of the *E. coli strains* were susceptible to meropenem, tigecycline, and colistin sulfate (Figure 3B). All *E. coli* strains from 2017–2019 were found 100% susceptible to meropenem, tigecycline, and colistin sulfate. Additionally, the AMR rate of AMP (44.23%), CIP (40.38%), TET (44.23%), and GEN (4.81%) was noted higher with a significant difference (*p < 0.05*) in 2017 than in other sampling years. Moreover, *E. coli* strains showed higher AMR to CTX (55.93%), SXT (68.64%), and AMK (3.39%) in 2018 with a significant difference (*p < 0.05*). However, none of the *E. coli* strain from 2017 and 2019 exhibited resistance to AMK and FOS (Figure 3C).

### 3.3. AMR characteristics of *Escherichia coli* strains isolated from different sources

**Figure 4:**
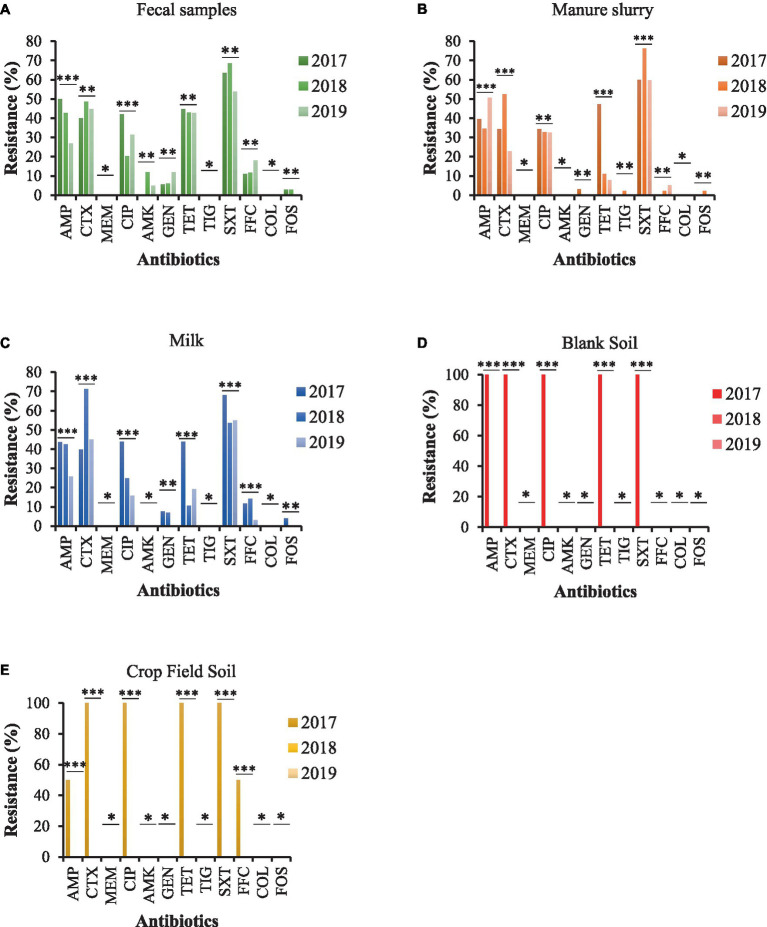
AMR rates of *E. coli* strains isolated from different sources. **(A)** fecal sample. **(B)** manure slurry from the storage tank. **(C)** raw milk. **(D)** blank soil. **(E)** crop field soil. AMP, ampicillin; CTX, cefotaxime; MEM, meropenem; CIP, ciprofloxacin; AMK, amikacin; GEN, gentamicin; TET, tetracycline; TIG, tigecycline; SXT, trimethoprim/sulfamethoxazole; FOS, fosfomycin; COL, colistin; FFC, florfenicol; **, indicate non-significant difference (*p* > 0.05); *, indicate not applicable; ***, indicate significant difference (*p* < 0.05).

Most of the *E. coli* strains from all samples were resistant to trimethoprim/sulfamethoxazole (SXT) and 100% susceptible to meropenem (MEM), tigecycline (TIG), and colistin sulfate (CS). Moreover, *E. coli* strains from fecal samples exhibited higher resistance to ampicillin (AMP), ciprofloxacin (CIP), and tetracycline (TET) in 2017 than other sampling years with a significant difference (Figure 4A). A similar trend was observed for *E. coli* strains isolated from milk and manure slurry (Figures 4B,C). Furthermore, *E. coli* strains isolated from blank and crop field soil in 2017 exhibited 100% resistant to CTX, CIP, TET, and SXT, while none of the strains isolated from 2018 and 2019 was resistant (Figures 4D,E). In addition, *E. coli* strains from crop field showed 50% resistance to AMP and florfenicol (FFC).

### 3.4. Drug resistance spectrum

**Figure 5:**
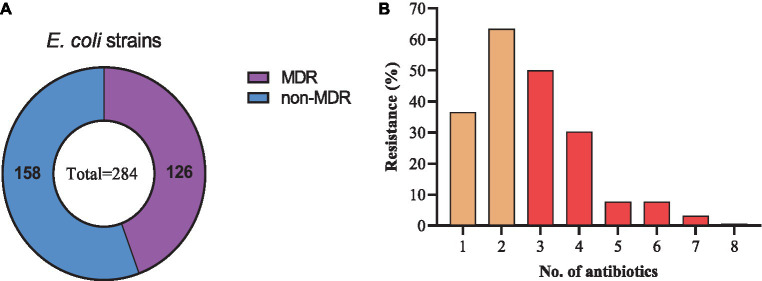
Drug-resistance spectrum of *E. coli* strains isolated from the dairy environment. **(A)** proportions of MDR and non-MDR strains. **(B)** Percentage resistance spectrum of 284 *E. coli* strains to 1 ~ 8 antibiotics.

Among the resistant strains, 126/284 (44.4%) were identified as multi-drug resistant (MDR) and 158/284 (55.6%) were recognized as non-MDR (Figure 5A). Most of the strains showed resistance to 2 antibiotics (63.38%), followed by 3 (50.0%), 1 (36.61%), 4 (30.28%), 5 and 6 (7.75% each), 7 (3.17%), and 8 (0.70%) antibiotics (Figure 5B). Moreover, diverse AMR patterns were recognized such as CTX + AMP, CTX + AMP + SXT, CTX + AMP + SXT + CIP, CTX + TET + SXT + CIP, CTX + AMP + SXT + CIP + GEN, CTX +AMP + SXT + CIP + TET + FFC, AMP + CTX + GEN + TET + SXT + FFC + FOS, and AMP + CTX + CIP + GEN + TET + SXT + FFC. Only 1 strain showed resistance to 8 antibiotics, AMP + CTX + CIP + AMK + GEN + TET + SXT + FFC (Table 1).

**Table 1:** The phenotypic drug resistance spectrum of *Escherichia coli* strains.

Antibiotic classes	Phenotypic resistance spectrum	No. of antibiotics	No. of strains
Sulfonamides	SXT	1	104
Cephalosporin + Penicillin	CTX + AMP	2	180
Cephalosporin + Penicillin + Sulfonamides	CTX + AMP + SXT	3	142
Cephalosporin + Penicillin + Sulfonamides + Quinolones	CTX + AMP + SXT + CIP	4	46
Cephalosporin + Tetracycline + Sulfonamides + Quinolones	CTX + TET + SXT + CIP	4	40
Cephalosporin + Penicillin + Sulfonamides + Quinolones + Aminoglycosides	CTX + AMP + SXT + CIP + GEN	5	22
Cephalosporin + Penicillin + Sulfonamides + Quinolones + Tetracycline + Amphenicol	CTX + AMP + SXT + CIP + TET + FFC	6	22
Penicillin + Cephalosporin + Aminoglycoside + Tetracycline + Sulfonamides + Amphenicol + Phosphonic	AMP + CTX + GEN + TET + SXT + FFC + FOS	7	5
Penicillin + Cephalosporin + Quinolones + Aminoglycoside + Tetracycline + Sulfonamides + Amphenicol	AMP + CTX + CIP + GEN + TET + SXT + FFC	7	4
Penicillin + Cephalosporin + Quinolones + Aminoglycosides + Tetracycline + Sulfonamides + Amphenicol	AMP + CTX + CIP + AMK + GEN + TET + SXT + FFC	8	2

AMP, ampicillin; CTX, cefotaxime; CIP, ciprofloxacin; GEN, gentamicin; TET, tetracycline; TIG, tigecycline; SXT, trimethoprim/sulfamethoxazole; FFC, florfenicol; AMK, Amikacin.

### 3.5. Detection of ARGs and correlation with phenotypic resistance

**Figure 6:**
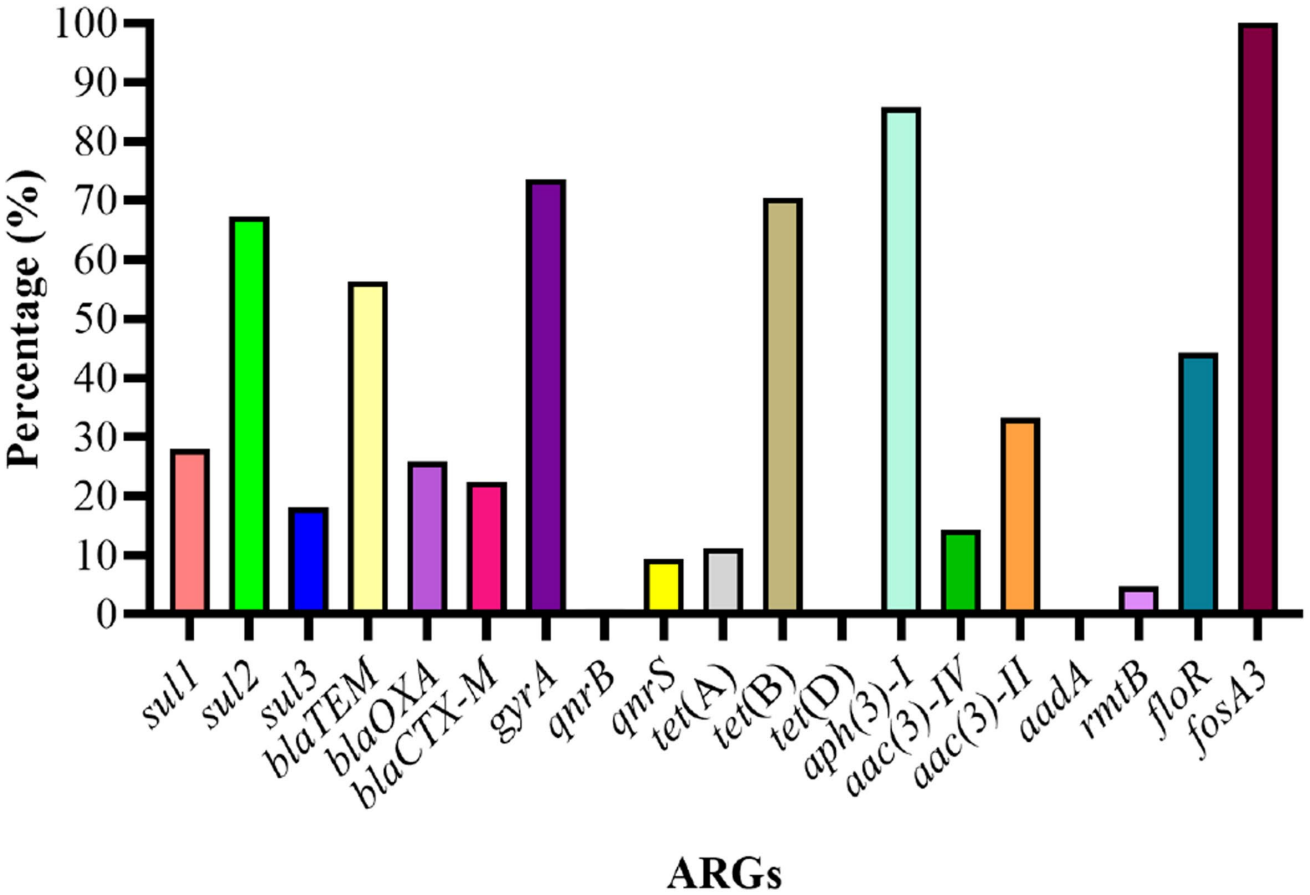
Percentage distribution of ARGs among *E. coli* strains.

The genotypic analysis was done by targeting 20 ARGs among 8 classes of antibiotics (mentioned in Table 1). Eighteen out of 20 ARGs were identified, and the prevalent genotypes included *sul2* (67.3%, sulfonamides), *bla_TEM_* (56.3%, beta-lactams), *gyrA* (73.6%, quinolones), *tet*(B) (70.4%, tetracycline’s), *aph*(3)-I (85.7%, aminoglycosides), *floR* (44.4%, amphenicol), and *fosA3* (100%, phosphonic). The percentage distribution of other ARGs identified was as follows: sulfonamides (*sul1*, 27.9%; *sul3*, 18.1%), β-lactams (*bla_OXA_*, 25.8%; *bla_CTX-M_*, 22.4%), aminoglycosides (*aac*(3)-IV, 14.3%; *aac*(3)-II, 33.3%; *aadA*, 0.00%; *rmtB*, 4.76%), quinolones (*qnrB*, 0.94%; *qnrS*, 9.43%), polymyxin (*pmrB*, 0.35%) and tetracycline’s (*tet*(A), 11.2%; *tet*(D), 0.00%) (Figure 6).

The correlation between phenotypic resistance and genotypic detection of ARGs was noted variable. For example, no strains showed resistance to colistin sulfate phenotypically but one strain was carrying the ARG upon genotypic analysis. Moreover, the number of strains carrying ARGs was noted higher as compared to phenotypic resistance among sulfonamides, beta-lactams, and aminoglycosides-resistant strains while the inverse was noted among quinolone, tetracycline, and amphenicol-resistant strains. However, the phenotypic and genotypic expression was observed 100% correlated for fosfomycin-resistant strains (Table 2).

**Table 2:** Percentage of resistant strains carrying ARGs and correlation with phenotypic resistance.

Antibiotic class	Resistance Phenotype	No. (%) of resistant strains (*n* = 338)	ARGs	Phenotypically resistant strains carrying ARGs		% difference in correlation
				No. (%)	Total/Class	
Sulfonamides	SXT	211 (62.4%)	*sul1*	59 (27.9)	239	13.3%
			*sul2*	142 (67.3)		
			*sul3*	38 (18.1)		
β-lactams	AMP + CTX	263 (77.8%)	*bla_TEM_*	148 (56.3)	275	4.56%
			*bla_OXA_*	68 (25.8)		
			*bla_CTX-M_*	59 (22.4)		
Quinolone	CIP	106 (31.4%)	*gyrA*	78 (73.6)	89	16.0%
			*qnrB*	1 (0.94)		
			*qnrS*	10 (9.43)		
Tetracycline’s	TET	98 (29.0%)	*tet*(A)	11 (11.2)	80	18.4%
			*tet*(B)	69 (70.4)		
			*tet*(D)	0 (0.00)		
Aminoglycosides	AMK + GEN	21 (6.21%)	*aph(3)-I*	18 (85.7)	29	38.1%
			*aac(3)-IV*	3 (14.3)		
			*aac(3)-II*	7 (33.3)		
			*aadA*	0 (0.00)		
			*rmtB*	1 (4.76)		
Amphenicol	FFC	27 (7.99%)	*flo*R	12 (44.4)	12	55.5%
Phosphonic	FOS	4 (1.18%)	*fosA3*	4 (100)	4	0.00%
Polymyxin	CS	0 (0.00%)	*pmrB*	1	1	N/A

### 3.6. Virulome gene analysis

**Figure 7:**
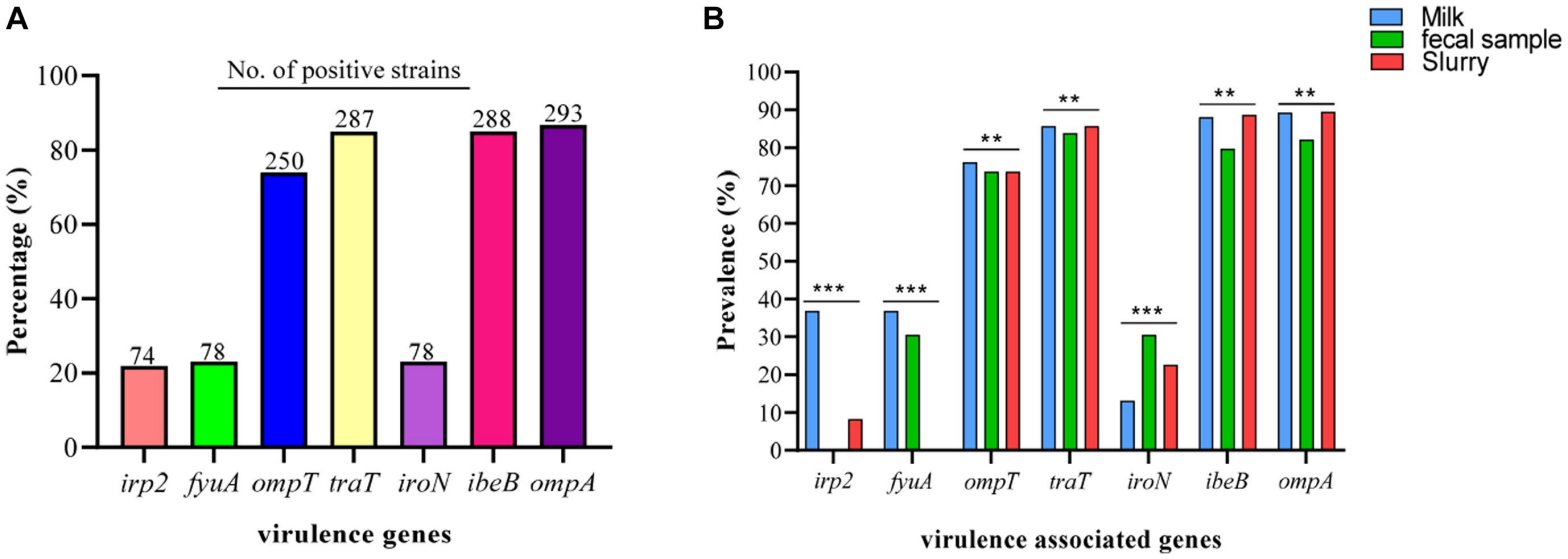
*Escherichia coli* strains carrying virulence-associated genes (VAGs). **(A)** Overall percentage and number of positive *E. coli* strains to carry VAGs. **(B)** Percentage distribution of VAGs among the *E. coli* strains isolated from different sources.

Among the VAGs, *ompA* was most prevalent (86.69%), followed by *ibeB* (85.0%), *traT* (84.91%), *ompT* (73.96%), *fyuA* (23.1%), *iroN* (23.1%), and *irp2* (21.9%) (Figure 7A). All of the *E. coli* strains carrying *the fyaA* gene were also carrying *the iroN* gene. Moreover, 93.59% of *E. coli* strains carrying *irp2* were also harboring *the fyuA* gene. VAGs such as *ompT*, *traT*, *iroN*, *ibeB*, and *ompA* were detected in *E. coli* strains from all sources while *irp2* and *fyuA* genes were not observed from manure slurry and fecal samples, respectively. However, both *irp2* and *fyuA* genes (36.9%, 31/84) were identified in strains of milk origin. Collectively, a higher percentage of VAGs was identified in strains of milk origin as compared to feces and slurry (Figure 7B).

### 3.7. Distribution of VAGs among phylogenetic groups

The phylogenetic analysis of 338 *E. coli* strains showed most of the strains belong to the B1 group (75.45%, 255/338), followed by A (18.34%, 62/338), C (2.96%, 10/338), D (1.18%, 4/338), E (1.18%, 4/338), and F (0.30%, 1/338). However, the phylogenetic group for 2 of the strains was not identified. The most prevalent VAGs among the various phylogenetic groups were as follows; B1 (*ompA*, 87.4%), A (*ibeB* and *ompA*, 88.7%), C (*traT* and *ompA*, 90.0%), D (*traT*, *ibeB*, and *ompA*, 100%), E (*traT*, 100%), and F (*traT*, 100%). Moreover, the percentage distribution of other VAGs among the phylogenetic groups is presented in Table 3.

**Table 3:** Distribution of VAGs in different phylogeny groups.

VAGs	No. Positive (%)					
	A (*n* = 62)	B1 (*n* = 255)	C (*n* = 10)	D (*n* = 4)	E (*n* = 4)	F (*n* = 1)
*fyuA*	21 (33.9)	52 (20.4)	2 (20.0)	3 (75.0)	0 (0.00)	0 (0.00)
*irp2*	19 (30.6)	50 (19.6)	2 (20.0)	3 (75.0)	0 (0.00)	0 (0.00)
*traT*	47 (75.8)	220 (86.3)	9 (90.0)	4 (100)	4 (100)	1 (100)
*ompT*	39 (62.9)	202 (79.2)	2 (20.0)	4 (100)	2 (50.0)	0 (0.00)
*iroN*	15 (24.2)	61 (23.9)	2 (20.0)	0 (0.00)	0 (0.00)	0 (0.00)
*ibeB*	55 (88.7)	221 (86.7)	7 (70.0)	4 (100)	0 (0.00)	0 (0.00)
*ompA*	55 (88.7)	223 (87.4)	9 (90.0)	4 (100)	1 (25.0)	0 (0.00)

## 4. Discussion

Antimicrobial resistance particularly in *the Enterobacteriaceae* family possesses a major threat to global public health. The present study isolated *E. coli* from the dairy environment, which serves as a reservoir of bacterial pathogens and ARGs and a source of spread of ARGs between the bacterial species *via* horizontal gene transfer and to humans *via* fecal contamination of drinking water and milk. The isolation rates of *E. coli* in this study were found similar to the findings of Sobur et al. (2019) who reported 75% prevalence of *E. coli* from dairy cattle and farm environment. Other studies conducted by Li et al. (2022) reported 84.6% *E. coli* isolation from fecal samples of cattle, chicken, and pigs while 34.4% was noted by Liu et al. (2021) from raw milk samples and 81.1% from raw cheese (Imre et al., 2022). Beattie et al. (2020) also reported a similar isolation rate of *E. coli* from dairy manure in the USA. It is also reported that the presence of *E. coli* in the dairy environment may be the cause of clinical mastitis in dairy cows (Su et al., 2016).

The antimicrobial susceptibility results showed higher resistance to SXT, followed by CTX, AMP, CIP, TET, and the least resistance to FFC, GEN, AMK, and FOS. These results are consistent with the findings of Peng et al. (2022) who isolated *E. coli* strains from pigs that were highly resistant to SXT (80.38%), AMP (92.86%), and TET (96.26%). A similar study conducted by Beattie et al. (2020) reported that *E. coli* strains from manure isolates showed higher resistance to AMP and CTX. However, a lower resistance rate to AMK and GEN was also noted by Lu et al. (2022) and Liu et al. (2021) respectively. We noted the *E. coli* strains were susceptible to the “last resort” antimicrobials such as MEM, TIG, and CS, which is consistent with the findings of a study conducted by Hu et al. (2019) in the human setting. Moreover, other studies conducted by Wang et al. (2021), Zou et al. (2021), and Ma et al. (2022) in animal settings also reported 100% susceptibility of MEM and TIG against *E. coli* strains. We noted no resistance to CS, which is consistent with a previous report that colistin resistance is decreasing in animal and human settings because of the CS ban in China (Wang et al., 2020). The percentage of MDR *E. coli* was noted at 44.4% in the current study, which is comparable with what was previously reported (54.4%) by Su et al. (2016) and lower than what was reported by Yu et al. (2020) in dairy milk. Another study conducted by Salinas et al. (2019) reported higher resistance to SXT, CIP, AMP, CTX, and TET by *E. coli* isolated from child and domestic animal origin, which is also consistent with current findings.

We identified 18 ARGs out of 20 belonging to different classes of antibiotics. The most prevalent ARGs were *sul2* (67.3%, sulfonamides), *bla_TEM_* (56.3%, beta-lactam), *gyrA* (73.6%, quinolones), *tet(B)* (70.4%, tetracycline’s), *aph(3)-I* (85.7%, aminoglycosides), *floR* (44.4%, amphenicol), and *fosA3* (100%, fosfomycin). Previous studies reported AMR in humans is linked to food animals raised for milk and meat purposes because of environmental contamination and drug residues (Bacanlı and Başaran, 2019; Pormohammad et al., 2019; Ma et al., 2021). The use of antimicrobial drugs in food animals also enhances the percentage of MDR bacteria and ARGs in human microbiota (Ma et al., 2022). Moreover, *E. coli* is also known to serve as donor bacteria for horizontal gene transfer within and between species (Oladeinde et al., 2019). Lima et al. (2020) highlighted the importance of animal manure and manure-substituted agriculture lands as a major source of antibiotic residues, ARGs, and AMR bacteria in the environment posing a potential threat to public health *via* horizontal gene transfer mechanisms with the help of mobile genetic elements such as plasmids, transposons, and integrons. Qian et al. (2018) detected 109 ARGs from the fresh manure of chicken, cattle, and pigs responsible for AMR to a class of antibiotics widely used in human and animal settings.

We investigated multiple VAGs in the isolated *E. coli* and most of the investigated VAGs (*ompA, ibeB, traT, ompT, fyuA, iroN, irp2*) belong to ExPEC, which may cause urinary tract infections in humans. VAGs are responsible for the production of virulence factors which play an important role in the pathogenicity of bacteria through multiple mechanisms such as adhesion, invasion, toxin production, and immune evasion (Kudva et al., 2020). Virulence genes investigated in the present study have various functions. *ompA* encodes for outer membrane protein A, *ibeB* is an invasion protein gene, *traT* encodes for complement resistance protein, *ompT* encodes for outer membrane protease protein, *fyuA* encodes for yersiniabactin receptor, *iroN* encodes for aerobactin receptor, and *irp2* gene encodes for iron-responsive element binding protein 2. Zhang et al. (2021) investigated similar VAGs in *E. coli* strains isolated from healthy waterfowls in Hainan, China. A study carried out by Raimondi et al. (2019) also identified similar VAGs in *E. coli* isolated from the feces of healthy individuals in Italy. Another study conducted by Khalifeh and Obaidat (2022) identified *the iroN* gene in *E. coli* strains from milk and fecal origin similar to the present study. In the current study, most of the *E. coli* strains were classified under phylogenetic group B1, which is consistent with the findings of Raimondi et al. (2019). These results suggest that the occurrence of ARGs and VAGs may vary by antimicrobial use and other unknown factors. This study also suggests regular monitoring of antimicrobial usage on dairy farms and proper manure treatment before disposal be ensured.

## 5. Conclusion

The present study identified multi-drug resistant *E. coli* strains carrying various ARGs and VAGs in the dairy environment, which may pose a potential threat to human, animal, and environmental health. Moreover, all of the *E. coli* strains were susceptible to meropenem, tigecycline, and colistin sulfate, which may be considered as critical antibiotics for therapeutic purposes in human and animal settings. Given the widespread distribution of AMR in the dairy environment, it is a potential reservoir of transferring ARGs genes to humans *via* various direct and indirect gene transfer mechanisms. This prudent the use of antibiotics on dairy farms, proper manure treatment, and enhancement of sanitation, especially in milk processing and transportation, are necessary to reduce the risk to food safety, public health, and environmental health.

## Data availability statement

The original contributions presented in the study are included in the article/Supplementary material, further inquiries can be directed to the corresponding author.

## Author contributions

MS: writing–original draft. ZH and XG: data curation and formal analysis. MT and RH: graphical representation of data. SW and RS: review and editing. XW and HZ: project administration. WP: conceptualization, supervision, project visualization, and funding acquisition. All authors contributed to the article and approved the submitted version.

## Funding

This work was supported by the National Key Research and Development Program (2016YFD0501305) and the Agricultural Science and Technology Innovation Program of the Chinese Academy of Agricultural Sciences (25-LZIHPS-03).

## Conflict of interest

The authors declare that the research was conducted in the absence of any commercial or financial relationships that could be construed as a potential conflict of interest.

## Publisher’s note

All claims expressed in this article are solely those of the authors and do not necessarily represent those of their affiliated organizations, or those of the publisher, the editors and the reviewers. Any product that may be evaluated in this article, or claim that may be made by its manufacturer, is not guaranteed or endorsed by the publisher.

## Footnotes

^1^ https://www.eucast.org

## References

[ref1] Afema J. A. Ahmed S. Besser T. E. Jones L. P. Sischo W. M. Davis M. A. (2018). Molecular epidemiology of dairy cattle-associated *Escherichia coli* carrying Bla CTX-M genes in Washington state. Appl. Environ. Microbiol. 84, e02430–e02417. doi: 10.1128/AEM.02430-17 29305512 PMC5835732

[ref2] Alfinete N. W. Bolukaoto J. Y. Heine L. Potgieter N. Barnard T. G. (2022). Virulence and phylogenetic analysis of enteric pathogenic *Escherichia coli* isolated from children with diarrhoea in South Africa. Int. J. Infect. Dis. 114, 226–232. doi: 10.1016/j.ijid.2021.11.017, PMID: 34775113

[ref3] Amézquita-López B. A. Soto-Beltrán M. Lee B. G. Yambao J. C. Quiñones B. (2018). Isolation, genotyping and antimicrobial resistance of Shiga toxin-producing *Escherichia coli*. J. Microbiol. Immunol. Infect. 51, 425–434. doi: 10.1016/j.jmii.2017.07.004 28778595

[ref4] Bacanlı M. Başaran N. (2019). Importance of antibiotic residues in animal food. Food Chem. Toxicol. 125, 462–466. doi: 10.1016/j.fct.2019.01.033 30710599

[ref5] Beattie R. E. Bakke E. Konopek N. Thill R. Munson E. Hristova K. R. (2020). Antimicrobial resistance traits of *Escherichia coli* isolated from dairy manure and freshwater ecosystems are similar to one another but differ from associated clinical isolates. Microorganisms 8:747. doi: 10.3390/microorganisms8050747, PMID: 32429352 PMC7284991

[ref6] Cheng J. Qu W. Barkema H. W. Nobrega D. B. Gao J. Liu G.. (2019). Antimicrobial resistance profiles of 5 common bovine mastitis pathogens in large Chinese dairy herds. J. Dairy Sci. 102, 2416–2426. doi: 10.3168/jds.2018-15135, PMID: 30639013

[ref7] Cookson A. L. Marshall J. C. Biggs P. J. Rogers L. E. Collis R. M. Devane M.. (2022). Whole-genome sequencing and virulome analysis of *Escherichia coli* isolated from New Zealand environments of contrasting observed land use. Appl. Environ. Microbiol. 88, e00277–e00222. doi: 10.1128/aem.00277-22 35442082 PMC9088250

[ref8] Dadgostar P. (2019). Antimicrobial resistance: implications and costs. Infect. Drug Resist. 12, 3903–3910. doi: 10.2147/IDR.S234610, PMID: 31908502 PMC6929930

[ref9] Graham D. W. Bergeron G. Bourassa M. W. Dickson J. Gomes F. Howe A.. (2019). Complexities in understanding antimicrobial resistance across domesticated animal, human, and environmental systems. Ann. N. Y. Acad. Sci. 1441, 17–30. doi: 10.1111/nyas.14036, PMID: 30924539 PMC6850694

[ref10] He T. Wang R. Liu D. Walsh T. R. Zhang R. Lv Y.. (2019). Emergence of plasmid-mediated high-level tigecycline resistance genes in animals and humans. Nat. Microbiol. 4, 1450–1456. doi: 10.1038/s41564-019-0445-2, PMID: 31133751

[ref11] Hogan J. Smith K. (1992). Using bulk tank milk cultures in a dairy practice. Workshop mastitis microbiology diagnostics, National Mastitis Council, Arlington.

[ref12] Hu F. Guo Y. Yang Y. Zheng Y. Wu S. Jiang X.. (2019). Resistance reported from China antimicrobial surveillance network (CHINET) in 2018. Eur. J. Clin. Microbiol. Infect. Dis. 38, 2275–2281. doi: 10.1007/s10096-019-03673-1, PMID: 31478103

[ref13] Hu B. Yang X. Liu Q. Zhang Y. Jiang D. Jiao H.. (2022). High prevalence and pathogenic potential of Shiga toxin-producing *Escherichia coli* strains in raw mutton and beef in Shandong, China. Curr. Res. Food Sci. 5, 1596–1602. doi: 10.1016/j.crfs.2022.08.021, PMID: 36161222 PMC9493282

[ref14] Imre K. Ban-Cucerzan A. Herman V. Sallam K. I. Cristina R. T. Abd-Elghany S. M.. (2022). Occurrence, pathogenic potential and antimicrobial resistance of *Escherichia coli* isolated from raw Milk cheese commercialized in Banat region, Romania. Antibiotics 11:721. doi: 10.3390/antibiotics11060721, PMID: 35740128 PMC9220297

[ref15] Jara M. C. Frediani A. V. Zehetmeyer F. K. Bruhn F. R. P. Müller M. R. Miller R. G.. (2021). Multidrug-resistant hospital bacteria: epidemiological factors and susceptibility profile. Microb. Drug Resist. 27, 433–440. doi: 10.1089/mdr.2019.0209 32706621

[ref16] Khalifeh O. M. Obaidat M. M. (2022). Urinary tract virulence genes in extended-spectrum beta-lactamase *E. coli* from dairy cows, beef cattle, and small ruminants. Acta Trop. 234:106611. doi: 10.1016/j.actatropica.2022.106611, PMID: 35850234

[ref17] Kudva I. T. Cornick N. A. Plummer P. J. Zhang Q. Nicholson T. L. Bannantine J. P.. (2020). Virulence mechanisms of bacterial pathogens. Hoboken, NJ: John Wiley & Sons.

[ref18] Li F. Cheng P. Li X. Liu R. Liu H. Zhang X. (2022). Molecular epidemiology and colistin-resistant mechanism of *mcr*-positive and *mcr*-negative *Escherichia coli* isolated from animal in Sichuan province, China. Front. Microbiol. 13:8548. doi: 10.3389/fmicb.2022.818548 PMC9002323 35422787

[ref19] Lima T. Domingues S. Da Silva G. J. (2020). Manure as a potential hotspot for antibiotic resistance dissemination by horizontal gene transfer events. Vet. Sci. 7:110. doi: 10.3390/vetsci7030110, PMID: 32823495 PMC7558842

[ref20] Liu H. Meng L. Dong L. Zhang Y. Wang J. Zheng N. (2021). Prevalence, antimicrobial susceptibility, and molecular characterization of *Escherichia coli* isolated from raw milk in dairy herds in northern China. Front. Microbiol. 12:656. doi: 10.3389/fmicb.2021.730656 PMC8500479 34630355

[ref21] Lu Q. Zhang W. Luo L. Wang H. Shao H. Zhang T.. (2022). Genetic diversity and multidrug resistance of phylogenic groups B2 and D in InPEC and ExPEC isolated from chickens in Central China. BMC Microbiol. 22, 1–12. doi: 10.1186/s12866-022-02469-2 35180845 PMC8855568

[ref22] Ma F. Xu S. Tang Z. Li Z. Zhang L. (2021). Use of antimicrobials in food animals and impact of transmission of antimicrobial resistance on humans. Biosafety Health 3, 32–38. doi: 10.1016/j.bsheal.2020.09.004

[ref23] Ma J. Zhou W. Wu J. Liu X. Lin J. Ji X.. (2022). Large-scale studies on antimicrobial resistance and molecular characterization of *Escherichia coli* from food animals in developed areas of eastern China. Microbiol. Spectrum 10, e02015–e02022. doi: 10.1128/spectrum.02015-22 PMC9430128 35950758

[ref24] Manyi-Loh C. Mamphweli S. Meyer E. Okoh A. (2018). Antibiotic use in agriculture and its consequential resistance in environmental sources: potential public health implications. Molecules 23:795. doi: 10.3390/molecules23040795, PMID: 29601469 PMC6017557

[ref25] Murray C. J. Ikuta K. S. Sharara F. Swetschinski L. Aguilar G. R. Gray A.. (2022). Global burden of bacterial antimicrobial resistance in 2019: a systematic analysis. Lancet 399, 629–655. doi: 10.1016/S0140-6736(21)02724-0, PMID: 35065702 PMC8841637

[ref26] Oladeinde A. Cook K. Lakin S. M. Woyda R. Abdo Z. Looft T.. (2019). Horizontal gene transfer and acquired antibiotic resistance in *Salmonella enterica* serovar Heidelberg following in vitro incubation in broiler ceca. Appl. Environ. Microbiol. 85, e01903–e01919. doi: 10.1128/AEM.01903-19 31471306 PMC6821953

[ref27] Peng Z. Hu Z. Li Z. Zhang X. Jia C. Li T.. (2022). Antimicrobial resistance and population genomics of multidrug-resistant *Escherichia coli* in pig farms in mainland China. Nat. Commun. 13:1116. doi: 10.1038/s41467-022-28750-6, PMID: 35236849 PMC8891348

[ref28] Pormohammad A. Nasiri M. J. Azimi T. (2019). Prevalence of antibiotic resistance in *Escherichia coli* strains simultaneously isolated from humans, animals, food, and the environment: a systematic review and meta-analysis. Infect. Drug Resist. 12, 1181–1197. doi: 10.2147/IDR.S201324, PMID: 31190907 PMC6512575

[ref29] Qian X. Gu J. Sun W. Wang X.-J. Su J.-Q. Stedfeld R. (2018). Diversity, abundance, and persistence of antibiotic resistance genes in various types of animal manure following industrial composting. J. Hazard. Mater. 344, 716–722. doi: 10.1016/j.jhazmat.2017.11.020, PMID: 29154097

[ref30] Raimondi S. Righini L. Candeliere F. Musmeci E. Bonvicini F. Gentilomi G.. (2019). Antibiotic resistance, virulence factors, phenotyping, and genotyping of *E. coli* isolated from the feces of healthy subjects. Microorganisms 7:251. doi: 10.3390/microorganisms7080251, PMID: 31405113 PMC6722543

[ref31] Salinas L. Cárdenas P. Johnson T. J. Vasco K. Graham J. Trueba G. (2019). Diverse commensal *Escherichia coli* clones and plasmids disseminate antimicrobial resistance genes in domestic animals and children in a semirural community in Ecuador. Msphere 4, e00316–e00319. doi: 10.1128/mSphere.00316-19 31118304 PMC6531886

[ref32] Sarowska J. Futoma-Koloch B. Jama-Kmiecik A. Frej-Madrzak M. Ksiazczyk M. Bugla-Ploskonska G.. (2019). Virulence factors, prevalence and potential transmission of extraintestinal pathogenic *Escherichia coli* isolated from different sources: recent reports. Gut Pathogens 11, 1–16. doi: 10.1186/s13099-019-0290-0 30828388 PMC6383261

[ref33] Sharp J. L. Mobley C. Hammond C. Withington C. Drew S. Stringfield S.. (2012). A mixed methods sampling methodology for a multisite case study. J. Mixed Methods Res. 6, 34–54. doi: 10.1177/1558689811417133

[ref34] Sobur M. A. Sabuj A. A. M. Sarker R. Rahman A. T. Kabir S. L. Rahman M. T. (2019). Antibiotic-resistant *Escherichia coli* and *Salmonella* spp. associated with dairy cattle and farm environment having public health significance. Veterinary world 12, 984–993. doi: 10.14202/vetworld.2019.984-993, PMID: 31528022 PMC6702575

[ref35] Su Y. Yu C.-Y. Tsai Y. Wang S.-H. Lee C. Chu C. (2016). Fluoroquinolone-resistant and extended-spectrum β-lactamase-producing *Escherichia coli* from the milk of cows with clinical mastitis in southern Taiwan. J. Microbiol. Immunol. Infect. 49, 892–901. doi: 10.1016/j.jmii.2014.10.003, PMID: 25592882

[ref36] Thrusfield M. (2018). Veterinary epidemiology. Hoboken, NJ: John Wiley & Sons.

[ref37] Wang Y. Xu C. Zhang R. Chen Y. Shen Y. Hu F.. (2020). Changes in colistin resistance and mcr-1 abundance in *Escherichia coli* of animal and human origins following the ban of colistin-positive additives in China: an epidemiological comparative study. Lancet Infect. Dis. 20, 1161–1171. doi: 10.1016/S1473-3099(20)30149-3, PMID: 32505232

[ref38] Wang W. Yu L. Hao W. Zhang F. Jiang M. Zhao S.. (2021). Multi-locus sequence typing and drug resistance analysis of swine origin *Escherichia coli* in Shandong of China and its potential risk on public health. Front. Public Health 9:780700. doi: 10.3389/fpubh.2021.780700, PMID: 34926393 PMC8674453

[ref39] Yu Z. Wang J. Ho H. Wang Y. Huang S. Han R. (2020). Prevalence and antimicrobial-resistance phenotypes and genotypes of *Escherichia coli* isolated from raw milk samples from mastitis cases in four regions of China. J. Global Antimicrobial Resist. 22, 94–101. doi: 10.1016/j.jgar.2019.12.016, PMID: 31887413

[ref40] Zhang S. Chen S. Rehman M. U. Yang H. Yang Z. Wang M.. (2021). Distribution and association of antimicrobial resistance and virulence traits in *Escherichia coli* isolates from healthy waterfowls in Hainan, China. Ecotoxicol. Environ. Safety 220:112317. doi: 10.1016/j.ecoenv.2021.112317 34049228

[ref41] Zhao X. Lv Y. Adam F. E. A. Xie Q. Wang B. Bai X.. (2021). Comparison of antimicrobial resistance, virulence genes, phylogroups, and biofilm formation of *Escherichia coli* isolated from intensive farming and free-range sheep. Front. Microbiol. 12:699927. doi: 10.3389/fmicb.2021.699927, PMID: 34394043 PMC8362090

[ref42] Zou M. Ma P.-P. Liu W.-S. Liang X. Li X.-Y. Li Y.-Z.. (2021). Prevalence and antibiotic resistance characteristics of extraintestinal pathogenic *Escherichia coli* among healthy chickens from farms and live poultry markets in China. Animals 11:1112. doi: 10.3390/ani11041112, PMID: 33924454 PMC8070349

