# Supplementary material for: The emergence of multi-drug resistant and virulence gene carrying Escherichia coli strains in the dairy environment: a rising threat to the environment, animal, and public health
Source: Front Microbiol. 2023 Jul 13;14:1197579. doi: 10.3389/fmicb.2023.1197579 (PMC10382034; doi:10.3389/fmicb.2023.1197579)
Supplement: Supplementary file 1 [file Table_1.DOCX]

**Table 1.** Name of genes, primer sequences and sizes

| **Gene Name** | **Primers** | **Primer Sequences (5’----3’)** | **Annealing temperature (℃)** | **Amplicon size (bp)** | **References** |
| --- | --- | --- | --- | --- | --- |
| *fyuA* | *fyuA-F* | ATGATTAACCCCGCGACGGGAA | 62 | 786 | (Su et al., 2016) |
|  | *fyuA-R* | CGCAGTAGGCACGATGTTGTA |  |  |  |
| *irp2* | *irp2-F* | CTGATGAACTCACTCGCTATCC | 62 | 440 | (Bernreiter-Hofer et al., 2021) |
|  | *irp2-R* | AGCATCTCCTGGCTCTGCTC |  |  |  |
| *traT* | *traT-F* | GGTGTGGTGCGATGAGCACAG | 64 | 290 | (Zhang et al., 2021) |
|  | *traT-R* | CACGGTTCAGCCATCCCTGAG |  |  |  |
| *ompT* | *ompT-F* | ATATCAAAGGCTGGCTCCTCA | 60 | 373 | (Lu et al., 2022) |
|  | *ompT-R* | TAACCTGCATTGACTGCAACG |  |  |  |
| *ibeB* | *ibeB-F* | GTTCTCACTCAGCCAGAACG | 58 | 1172 | (Zhang et al., 2021) |
|  | *ibeB-R* | CATCCAGCACTTCCAGATAAC |  |  |  |
| *iroN* | *iroN-F* | AATCCGGCAAAGAGACGAACCGCCT | 64 | 553 | (Su et al., 2016) |
|  | *iroN-R* | GTTCGGGCAACCCCTGCTTTGACTTT |  |  |  |
| *ompA* | *ompA-F* | AGCTATCGCGATTGCAGTG | 59 | 919 | (Lu et al., 2022) |
|  | *ompA-R* | GGTGTTGCCAGTAACCGG |  |  |  |

**Table 2.** MIC breakpoints of different antibiotics

| MIC breakpoints mg/L | | |
| --- | --- | --- |
| **Antibiotics** | **Sensitive (S)** ≤ | **Resistant（R）>** |
| Ampicillin | 8 | 8 |
| Cefotaxime | 1 | 2 |
| Meropenem | 2 | 8 |
| Ciprofloxacin | 0.25 | 0.5 |
| Gentamicin | 2 | 2 |
| Amikacin | 8 | 8 |
| Tetracycline | 4 | 16 |
| Tigecycline | 0.5 | 0.5 |
| Florfenicol | 8 | 8 |
| Colistin | 2 | 2 |
| Trimethoprim-Sulfamethoxazole | 2 | 4 |
| Fosfomycin | 32 | 32 |

No intermediate breakpoints for following antibiotics found in EUCAST guideline;

**Table 3.** Antibiotic resistant genes, primer sequences and sizes

| **Gene Name** | **Primers** | **Primer Sequences (5’----3’)** | **Annealing temperature (℃)** | **Amplicon size (bp)** | **References** |
| --- | --- | --- | --- | --- | --- |
| *Sulfonamides* | *sul1* | ***F:*** GTGACGGTGTTCGGCATTCT | 58 | 779 | (Ma et al., 2022) |
|  |  | ***R:*** TCCGAGAAGGTGATTGCGCT |  |  |  |
|  | *sul2* | ***F:*** CATCATTTTCGGCATCGTC | 63 | 793 |  |
|  |  | ***R:*** TCTTGCGGTTTCTTTCAGC |  |  |  |
|  | *sul3* | ***F:*** AGATGTGATTGATTTGGGAGC | 57 | 443 |  |
|  |  | ***R:*** TAGTTGTTTCTGGATTAGAGCCT |  |  |  |
| *β-lactams* | *bla_OXA_* | ***F:*** ATATCTCTACTGTTGCATCTCC | 58 | 620 | (Liu et al., 2021) |
|  |  | ***R:*** AAACCCTTCAAACCATCC |  |  |  |
|  | *bla_CTX-M_* | ***F:*** CGCTTTGCGATGTGCAG | 58 | 551 |  |
|  |  | ***R:*** ACCGCGATATCGTTGGT |  |  |  |
|  | *bla_TEM_* | ***F:*** CAGAAACGCTGGTGAAAGTA | 55 | 719 |  |
|  |  | ***R:*** ACTCCCCGTCGTGTAGATAA |  |  |  |
| *Tetracycline* | *tet(A)* | ***F:*** CACTATGGCATTCTGCTGGC | 60 | 949 |  |
|  |  | ***R:*** CATAGATCGCCGTGAAGAGG |  |  |  |
|  | *tet(B)* | ***F:*** CTCAGTATTCCAAGCCTTTG | 59 | 436 |  |
|  |  | ***R:*** CTAAGCACTTGTCTCCTGTT |  |  |  |
|  | *tet(D)* | ***F:*** GCAAACCATTACGGCATTCT | 59 | 546 |  |
|  |  | ***R:*** GATAAGCTGCGCGGTAAAAA |  |  |  |
| *Quinolones* | *gyrA* | ***F:*** GGTGACGTAATCGGTAAATA | 52 | 810 | (Zhang et al., 2021) |
|  |  | ***R:*** ACCATGGTGCAATGCCACCA |  |  |  |
|  | *qnrB* | ***F:*** GATCGTGAAAGCCAGAAAGG | 56 | 417 |  |
|  |  | ***R:*** ACGATGCCTGGTAGTTGTCC |  |  |  |
|  | *qnrS* | ***F:*** ACGACATTCGTCAACTGCAA | 54 | 469 |  |
|  |  | ***R:*** TAAATTGGCACCCTGTAGGC |  |  |  |
| *Aminoglycosides* | *aph(3)-I* | ***F:*** ATGGGCTCGCGATAATGTC | 50 | 600 | (Yu et al., 2020) |
|  |  | ***R:*** CTCACCGAGGCAGTTCCAT |  |  |  |
|  | *aac(3)-IV* | ***F:*** CTTCAGGATGGCAAGTTGGT | 60 | 286 | (Zhang et al., 2021) |
|  |  | ***R:*** TCATCTCGTTCTCCGCTCAT |  |  |  |
|  | *aac(3)-II* | ***F:*** ACTGTGATGGGATACGCGTC | 60 | 237 |  |
|  |  | ***R:*** CTCCGTCAGCGTTTCAGCTA |  |  |  |
|  | *aadA* | ***F:*** TGATTTGCTGGTTACGGTGAC | 55 | 284 | (Yu et al., 2020) |
|  |  | ***R:*** CGCTATGTTCTCTTGCTTTTG |  |  |  |
|  | *rmtB* | ***F:*** GCTTTCTGCGGGCGATGTAA | 52 | 173 | (Zhang et al., 2021) |
|  |  | ***R:*** ATGCAATGCCGCGCTCGTAT |  |  |  |
| *Amphenicol* | *floR* | ***F:*** GTCGAGAAATCCCATGAGTTCA | 57 | 1645 |  |
|  |  | ***R:*** CAGACAGGATACCGACATTCAC |  |  |  |
| *Phosphonic* | *fosA3* | ***F:*** GCGTCAAGCCTGGCATTT | 63 | 282 |  |
|  |  | ***R:*** GCCGTCAGGGTCGAGAAA |  |  |  |
| *Polymyxin* | *pmrB* | ***F:*** ATAAGCTGAAACGGATGGC | 60 | 1312 | (Huang et al., 2017) |
|  |  | ***R:*** CATAATAATCAGGGCGAAAGT |  |  |  |

**Table 4.** Primers information used for phylogenetic group and results interpretation

| **PCR amplification** | | | | |  | **Results Interpretation** | | | | | |
| --- | --- | --- | --- | --- | --- | --- | --- | --- | --- | --- | --- |
| **PCR** | **Target Gene** | **Primer name** | **Sequences（5'-3'）** | **Size (bp）** | **References** | **Quadruplex PCR** | | | | **Phylogenetic group** | **Next move** |
| Quadruple PCR | *chuA* | ChuA:F | ATGGTACCGGACGAACCAAC | 288 | (Clermont et al., 2013) | ***arpA*** | ***chuA*** | ***yjaA*** | ***TspE4.C2*** |  |  |
|  |  | ChuA:R | TGCCGCCAGTACCAAAGACA |  |  | P | N | N | N | A | N/A |
|  | *yjaA* | YjaA:F | CAAACGTGAAGTGTCAGGAG | 211 |  | P | N | N | P | B1 | N/A |
|  |  | YjaA:R | AATGCGTTCCTCAACCTGTG |  |  | N | P | N | N | F | N/A |
|  | *tspE4C2* | TspE4.C2:F | CACTATTCGTAAGGTCATCC | 152 |  | N | P | P | N | B2 | N/A |
|  |  | TspE4.C2:R | AGTTTATCGCTGCGGGTCGC |  |  | N | P | P | P | B2 | N/A |
|  | *arpA* | AceK:F | AACGCTATTCGCCAGCTTGC | 400 |  | N | P | N | P | B2 | N/A |
|  |  | ArpA1:R | TCTCCCCATACCGTACGCTA |  |  | P | N | P | N | A/C | PCR using group C primers, If C+, then C, otherwise A |
| Group E | *arpA* | ArpAgpE:F | GATTCCATCTTGTCAAAATATGCC | 301 |  | P | P | N | N | D/E | PCR using group E primers, If E+, then E, otherwise D |
|  |  | ArpAgpE:R | GAAAAGAAAAAGAATTCCCAAGAG |  |  | P | P | N | P | D/E | PCR using group E primers, If E+, then E, otherwise D |
| Group C | *trpA* | trpAgpC:F | AGTTTTATGCCCAGTGCGAG | 219 |  |  |  |  |  |  |  |
|  |  | trpAgpC:R | TCTGCGCCGGTCACGCCC |  |  |  |  |  |  |  |  |
| Internal control | *trpA* | trpBA:F | CGGCGATAAAGACATCTTCAC | 489 |  |  |  |  |  |  |  |
|  |  | trpBA:R | GCAACGCGGCCTGGCGGAAG |  |  |  |  |  |  |  |  |


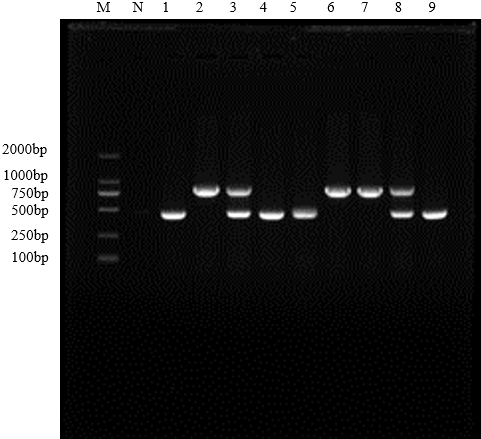

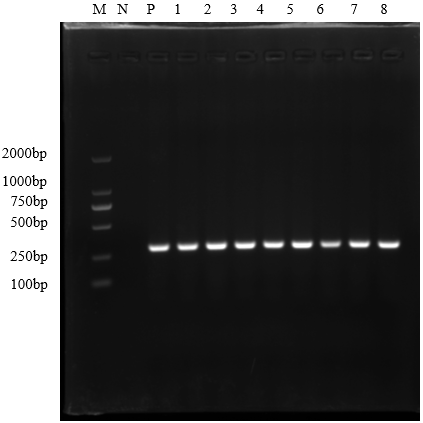

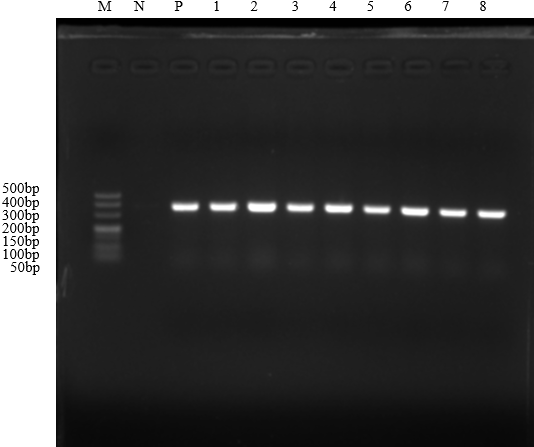


**(A)**

**(B)**

**(C)**


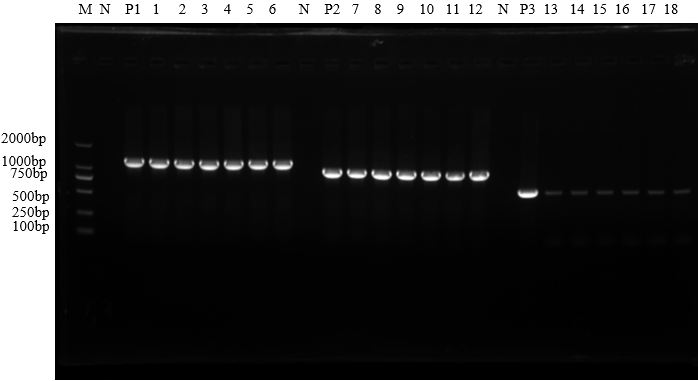


**(D)**

**Figure 1.** Agarose gel electrophoresis (AGE) of amplified PCR products of virulence genes; M= DNA Marker, N= Negative control, P= Positive Control **(A)** 1~9: AGE of *irp2* and *fyuA* gene positive isolates **(B)** 1~8: *traT* gene positive isolates **(C)** 1~8: *ompT* gene positive isolates **(D)** 1~6，7~12 and 13~18: AGE results of *ibeB, ompA,* and *iroN* genes respectively

**Figure S2.** Agarose gel electrophoresis of 16S rRNA gene amplified PCR product of *E. coli* isolates; M= Marker, 1= Positive control, 2-24= Positive isolates


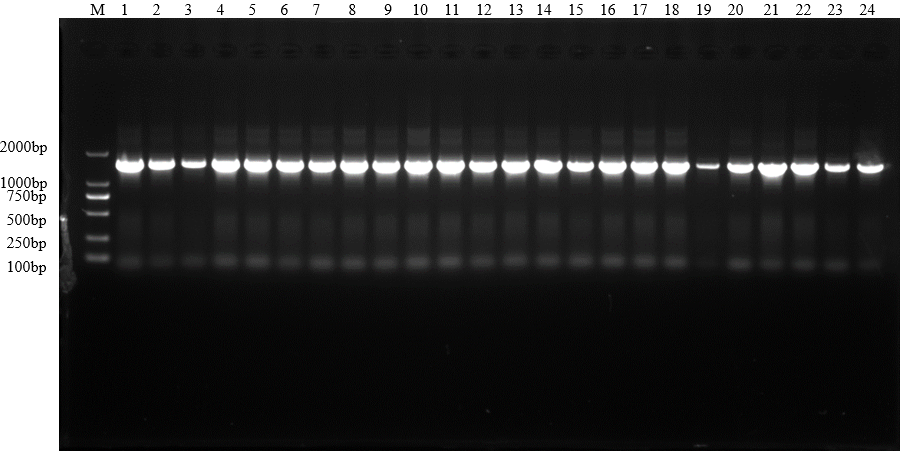


**
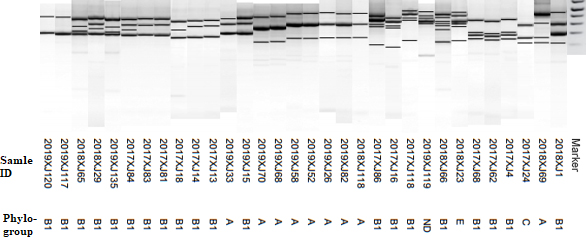
**

**Figure S3.** Representative diagram of amplified PCR product of *E. coli* strains on agarose gel, ND= non type-able
